# Microbial dynamics and *Pseudomonas* natural product production in milk and dairy products

**DOI:** 10.1039/d4np00074a

**Published:** 2025-03-03

**Authors:** Ina Wasmuth, Christina Warinner, Pierre Stallforth

**Affiliations:** a Department of Paleobiotechnology, Leibniz Institute for Natural Product Research and Infection Biology, Hans Knöll Institute 07745 Jena Germany christina_warinner@eva.mpg.de pierre.stallforth@leibniz-hki.de; b Department of Archaeogenetics, Max Planck Institute for Evolutionary Anthropology 04103 Leipzig Germany; c Associated Research Group of Archaeogenetics, Leibniz Institute for Natural Product Research and Infection Biology, Hans Knöll Institute 07745 Jena Germany; d Institute of Organic Chemistry and Macromolecular Chemistry, Friedrich Schiller University Jena 07743 Jena Germany; e Faculty of Biological Sciences, Institute of Microbiology, Friedrich Schiller University Jena 07743 Jena Germany; f Department of Anthropology, Harvard University Cambridge MA 02138 USA

## Abstract

Covering: 2000 up to the first half of 2024

Milk and its derived dairy products have long been integral to the human diet, with evidence of consumption dating back over 9000 years. Milk's high nutritional value renders dairy products an important element of human diet while also offering a fertile environment for microbial growth. Beneficial microorganisms in dairy products are often associated with biogenic and probiotic effects, whereas spoilage or pathogenic microorganisms can pose health risks. Fermentation is a key method to preserve milk. Whereas dairying practices in most parts of the world have been highly altered by industrialization over the past century, nomadic pastoralists in Mongolia notably retain a rich tradition of household-level dairy fermentation that has been practiced since 3000 BC. Milk-associated microorganisms produce a vast number of low molecular weight natural products that can mediate beneficial and detrimental interactions. Bacteria of the genus *Pseudomonas* are found in traditional Mongolian dairy products and are common contaminants in commercial dairy products, and they can strongly impact the quality and shelf-life of dairy products. These bacteria are well known for their ability to produce a variety of secondary metabolites, including nonribosomal (lipo)peptides, which are both structurally and functionally diverse. Lipopeptides can have antimicrobial properties, act as quorum sensing molecules, and contribute to biofilm formation due to their amphiphilic nature. Although often associated with spoilage, some of these natural products can also exhibit positive effects with potential beneficial applications in the dairy industry. This review aims to provide a comprehensive overview of the interplay between culinary fermentation and the production and activities of microbial-derived natural products.

## Abstract 2

This review focuses on the role of microorganisms and their natural products in milk and dairy products.

## Graphical abstract

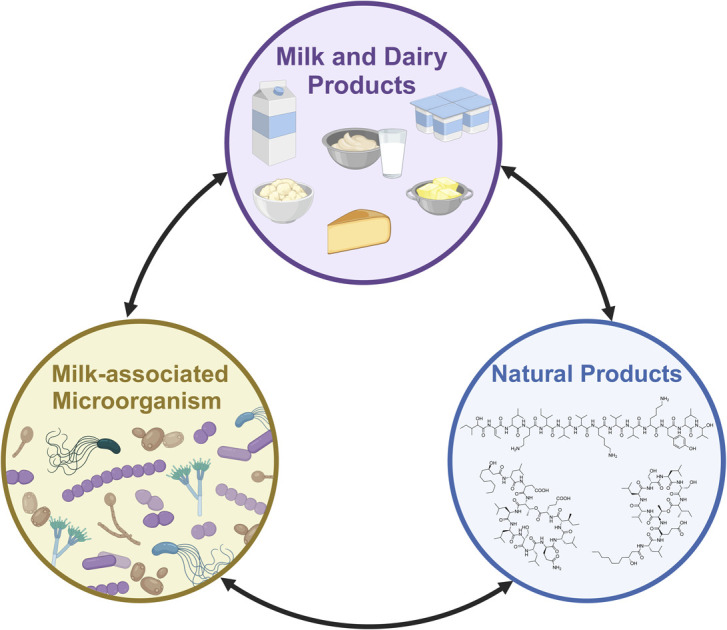

## 1 Introduction

The consumption of milk and dairy products has a deep history in human nutrition and began with the Neolithic domestication of dairy livestock in the Near East.^1,2^ Sheep and goats were first domesticated around 9300–8200 BC, followed by cattle around 8500 BC,^3–6^ and by the 7 millennium BC, animal milk had become an integral part of human diets at sites in Anatolia and the Levant.^1^ Dairy technologies spread throughout western Eurasia^7,8^ and Africa^9,10^ over the next three millennia, and by the Early Bronze Age dairy pastoralism had also expanded deep into eastern Eurasia, reaching Mongolia by 3000 BC^11–13^ and China by 2000 BC.^14^

Mongolia's rich dairying traditions provide unique insights into prehistoric dairying practices and the evolutionary history and biodiversity of traditional microbial ferments. Whereas dairying practices in most other parts of the world have been highly altered by industrialization over the past century, nomadic pastoralists in Mongolia continue to engage in a robust tradition of household-level dairy fermentation that has been practiced – without major interruption – for 5000 years.^15–17^ Traditional fermentation techniques in Mongolia are used to preserve milk and transform it into a wide variety of dairy products. Hence, we chose Mongolian dairying traditions to exemplify both techniques specific to that region as well as general concepts.

Due to its high nutrient content, near-neutral pH (pH = 6.63 ± 0.08)^18^ and high water activity (*a*_w_ = 0.99),^19^ milk serves as an ideal growth medium for many microorganisms. Lactic acid bacteria (LAB) as well as yeasts are key players in the fermentation of milk.^20,21^ These microbes may be present in the milk microbiota of the lactating animal, may be unintentionally introduced from environmental sources such as milking equipment and fermentation utensils, or may be intentionally added as starter cultures.^20,22^ Microbial interactions during fermentation can be both mutualistic and antagonistic, thereby influencing the fermentation process as well as the quality and safety of the products.^23^

The diverse microbiota present in raw milk and their role in fermentation, as well as their interaction and influence on milk quality, safety, and spoilage potential, have been extensively reviewed in the last decade.^20,24–27^ These reviews address different aspects of the composition of milk microbiota, how environmental and host factors shape milk microbiota, and how the milk microbiota influences the interaction between mother and offspring,^24,26^ as well as the benefits of heat treatment on reducing unwanted microorganisms to improve the quality of milk and dairy products.^27^

Today, the spoilage potential of milk is mainly attributed to psychrotrophic (cold-adapted) bacteria such as *Pseudomonas* and *Acinetobacter*,^20^ which can multiply under refrigeration at low temperatures and produce extracellular heat-resistant proteases and lipases^28,29^ that effectively reduce the shelf life of milk and dairy products.^30^ Numerous review articles on psychrotrophic bacteria and other spoilage microorganisms have discussed their occurrence and effects in dairy products.^30–33^

Common psychrotrophic dairy-associated bacteria such as *Pseudomonas* are noteworthy for their ability to produce a variety of secondary metabolites, including nonribosomal peptides (NRPs), polyketides (PKs), and other bioactive compounds.^34^ These metabolites play an important role in the ecological niche adaptation of these bacteria, ensuring their survival and enabling interactions with other microorganisms.^35,36^ In particular, nonribosomal peptide synthetase (NRPS) genes responsible for the production of cyclic and linear lipopeptides are highly represented in *Pseudomonas* genomes.^37^ There are now numerous reviews detailing the structural diversity of lipopeptides, especially those from *Pseudomonas* species, as well as their classification into different families and functional properties. These papers also highlight the diverse biological activities and ecological roles of lipopeptides from *Pseudomonas*, including their antimicrobial properties and functions in biofilm formation.^35,36,38–43^ However, none of these reviews address the production of these natural products in milk, even though nonribosomal peptides such as linear or cyclic lipopeptides in milk could potentially have beneficial effects on the microbial community and the preservation of milk and dairy products.

Overviews of the general role of *Pseudomonas* species and psychrotrophic microorganisms in dairy spoilage, as well as their antimicrobial properties, functions in biofilm formation, and quorum sensing capabilities are given elsewhere.^30–33,44^ However, little is known regarding the role of nonribosomal peptides such as lipopeptides in milk. This review aims to fill this gap by focusing on the production and biological activity of microbially derived natural products in milk. A particular focus is placed on the characterization of nonribosomal peptides and lipopeptides from *Pseudomonas* species that are commonly found in milk. Their potential beneficial effects on the dairy microbial community and the preservation of milk and dairy products will be discussed. This review offers useful insights into the relationship between traditional dairy pastoralism, the microbiological study of Mongolian dairy products, and microbially-derived natural products in dairy systems.

## 2 Anthropological perspective on dairy pastoralism in Mongolia

Mongolia is known for its rich nomadic heritage and unique culinary traditions centered around dairying and mobile pastoralism. Dairy pastoralism has a long and rich history in Mongolia, dating back to the Early Bronze Age approximately 5000 years ago with the arrival of the Afanasievo, an archaeological culture whose genetic origins can be traced to nomadic herders in the North Caucasus region of present-day Russia.^7,11–13^ The Afanasievo introduced a form of mobile dairy pastoralism to western and central Mongolia that was based on the milking of domesticated sheep, goats, and cattle, and by the Late Bronze Age dairy pastoralism had spread to become the main subsistence strategy practiced throughout nearly all of Mongolia. Horses, camels, yaks, and reindeer were later added as regionally important dairy livestock during the Iron Age and medieval periods,^11,45,46^ making Mongolia the country with the most milked animal species in the world. Dairying remains one of the most important economic activities in Mongolia today and represents the main livelihood for nearly one third of the population.^47,48^

**Fig. 1:**
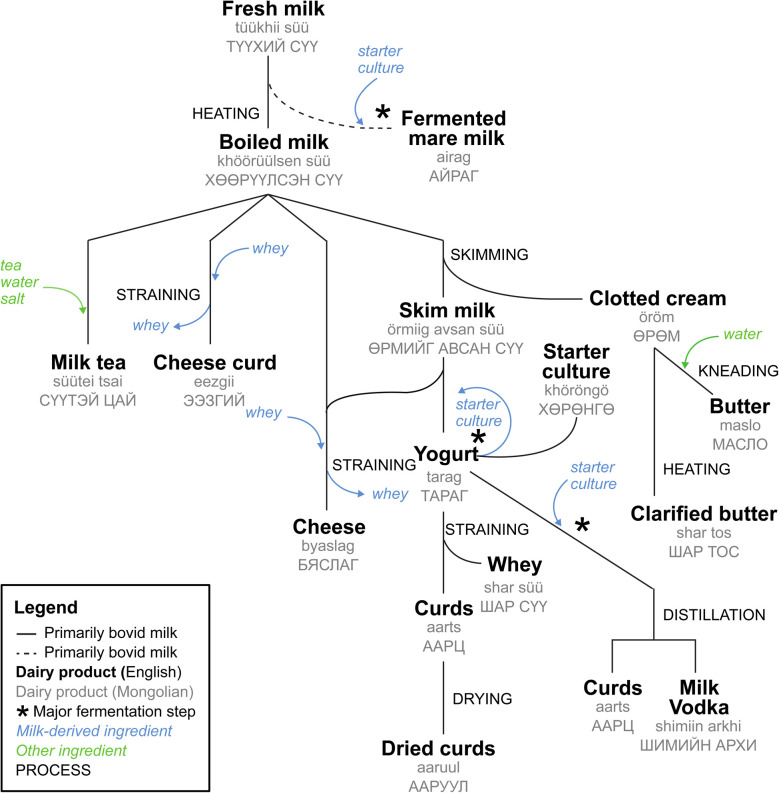
Process of producing typical Mongolian dairy products.

Over millennia, Mongolians have developed distinctive techniques to preserve and transform milk through fermentation into a variety of long-lasting and nutrient-rich products including yogurt (*tarag*), curds and cheeses (*aarts*, *aaruul*, *eezgii*, and *byaslag*), butters and clotted cream (*maslo*, *shar tos*, and *öröm*), and alcoholic beverages (*airag* and *shimiin arkhi*) (Fig. 1).^11^ The impressive range of fresh, fermented, processed, and distilled dairy products are produced using the milk of different livestock.^49^ The composition of dairy livestock herds varies regionally, with reindeer in the far north, cattle and yaks (as well as cattle-yak hybrids known as *khainag*) in the north, and camels in the south, while sheep, goats, and horses are generally found throughout Mongolia. Herders typically raise 1–5 species of livestock, and maintaining mixed herds allows greater exploitation of milk, meat, wool, leather, and hide, as well as labor and traction, upon which herders are dependent for managing their herds and moving to new pasture land.^11,15,50,51^

Among the most popular traditional Mongolian milk products is *airag* (alcoholic fermented mare's milk, in other regions also known as *koumiss*).^52^ It is not heated and undergoes fermentation due to the addition of a starter culture and the presence of indigenous microflora, especially lactic acid bacteria (LAB) and yeast, resulting in a slightly carbonated, sour, and mildly (approx. 2%) alcoholic beverage. *Airag* is generally considered to have health-promoting properties and is also used for medical purposes in Mongolia. Beyond its macronutrients, fermented mare milk also contains trace elements, vitamins (*e.g.*, B12, K2), exopolysaccharides, and antimicrobial substances^52,53^ that may promote gut health and strengthen the immune system. Furthermore, *airag* has an antihypertensive effect, lowers cholesterol levels and blood pressure, and is also ascribed to have an anti-inflammatory and anti-cancer effect.^54^

## 3 Fermentation potential of milk-associated microorganisms

Milk-associated microorganisms play a crucial role in dairy fermentation. Within the context of nomadic pastoralists, these microbes may originate within or on the teat of the lactating animal or they may be introduced through milking and processing equipment or by intentionally adding a starter culture.^20,22^ As such, the microbial composition of milk and fermented products varies slightly among different pastoralist households, which contributes to the rich diversity of fermented products.^49^ Differences in microbial composition can be observed between traditional and modern dairy products. Modern dairy products are produced using standardized industrial processes and defined starter cultures, resulting in reduced microbial diversity. In contrast, traditional fermentation methods are often based on spontaneous fermentation by native microbes or back-slopping techniques, which preserve a broader range of microbial communities.

A shared feature of milk-associated microbes is that they excrete a set of lipolytic, proteolytic, and amino acid-converting enzymes^55^ that improve the digestibility of milk and generate a range of beneficial metabolites (biogenic effect). These enzymatic and chemical transformations contribute to the characteristic flavors, textures, and nutritional profiles of fermented dairy products while also facilitating their preservation.^53^ Some milk-associated microorganisms are also able to produce vitamins such as folic acid^53^ (vitamin B9), which has health-promoting properties and is crucial for fetal spinal development during pregnancy,^56–58^ or have an otherwise unspecified general positive effect on the host (probiotic effect).^59^

### 3.1 Lactic acid bacteria (LAB)

**Fig. 2:**
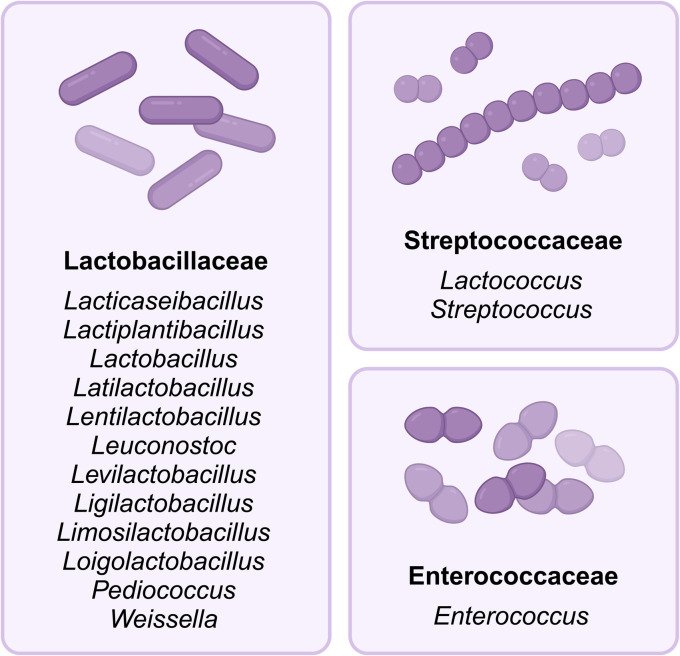
Overview of lactic acid bacteria (LAB) in raw milk. Created with BioRender.

Dairy-associated LAB species of the Enterococcaceae (*Enterococcus*), Lactobacillaceae (*Lacticaseibacillus*, *Lactiplantibacillus*, *Lactobacillus*, *Latilactobacillus*, *Lentilactobacillus*, *Leuconostoc*, *Levilactobacillus*, *Ligilactobacillus*, *Limosilactobacillus*, *Loigolactobacillus*, *Pediococcus*, and *Weissella*), and Streptococcaceae (*Lactococcus*, *Streptococcus*) families within the Lactobacillales order are a broad group of Gram-positive bacteria that are typically found in fresh milk and dominate the fermentation process of different dairy products (Fig. 2).^20,60^ They can be divided into two groups, mesophilic (20–30 °C) LAB and thermophilic (30–45 °C) LAB, which differ in their optimum growth temperature.^22^ Differences in ambient temperatures lead to a higher abundance of thermophilic LAB in milk and fermented dairy products in subtropical regions, while mesophilic LAB are more common in Western and Northern European countries.^22,53^

LAB are responsible for acidifying milk^20^ during fermentation by actively consuming lactose present in milk and converting it into lactic acid. Because few bacteria outside the Lactobacillales order grow well in an acidic environment, this has the added benefit of inhibiting the growth of many undesired and harmful bacteria.^61^ Lactic acid also aids in the denaturation and coagulation of milk proteins,^53^ leading to the thickening of milk and the formation of a curd. In European traditions, the addition of protein-hydrolyzing enzymes such as chymosin (the major enzyme in rennet) further facilitates the conversion of milk proteins, especially caseins, into peptides and amino acids. These amino acids serve as precursors for the formation of a variety of compounds, including aldehydes, alcohols, ketones, amines, acids, esters, and sulfur-containing compounds that contribute to the characteristic flavors of cheese.^53,55^

In addition to lactic acid, some LAB are also capable of producing ethanol and carbon dioxide through a process known as heterolactic fermentation. Heterolactic LAB include some members of the Lactobacillaceae family, most notably *Lactobacillus kefiri*, *Lacticaseibacillus paracasei*, *Lactiplantibacillus plantarum*, and *Leuconostoc mesenteroides*. Heterolactic LAB are important in the production of alcoholic dairy products in Mongolia, but are less utilized for this purpose in European dairying traditions. LAB that produce only lactic acid as the end product of fermentation are known as homolactic fermenters; most bacteria used in dairy production are homolactic LAB.

To accelerate the fermentation process, LAB can be enriched by back-slopping or by adding a starter culture. The thermophilic LAB *Streptococcus thermophilus* and *Lactobacillus delbrueckii* subsp. *bulgaricus* are the major components of yogurt starter cultures,^22^ while *lactis* and *cremoris* subspecies of the mesophilic LAB *Lactococcus lactis* are important starter culture components in the European cheese industry.^22,53^ The selection of appropriate starter cultures has the potential to improve the fermentation process and increase the overall quality of the end product.^57^ They are beneficial for standardizing fermented milk products but can also reduce the diversity of microorganisms in these products compared to those produced by spontaneous fermentation.

### 3.2 The effect of yeasts and mold in fermented milk

Eukaryotic microorganisms, such as yeasts and molds, are also present in many dairy products, although their presence substantially varies based on the type of milk, region, traditional practices, and methods used for fermentation.^52,53^ Overall, yeasts are typically found at higher abundance in dairy products than molds.^20^

Common yeast genera in raw milk are *Kluyveromyces*, *Saccharomyces*, *Pichia*, *Debaryomyces*, *Yarrowia*, and *Candida*.^20,21,62^ The wide variety of yeasts used in dairying have different physiological and biochemical properties that contribute to the quality and characteristics of fermented dairy products. Some yeasts are able to utilize citric, lactic, and succinic acids^63^ or have the ability to use lactose (*e.g.*, *Kluyveromyces marxianus*, *K. lactis*) or galactose (*e.g.*, *Saccharomyces unisporus*) as a carbon source.^53^ Yeasts of the genera *Saccharomyces* and *Kluyveromyces* are especially important for the production of alcohol and carbon dioxide in fermented dairy products like *airag* and *kefir*. Other yeasts have high proteolytic or lipolytic activity, like *Yarrowia lipolytica*, *Debaryomyces hansenii*, or *Geotrichum candidum*, and contribute to cheese ripening. Still, others can grow at low temperatures and are tolerant to high salt concentrations, low pH, and low water-activity (*a*_w_), which prevents spoilage.^20,63,64^

Molds change the texture and structure of dairy products through processes of proteolysis and lipolysis, which also alter aroma and taste, and they play a major role in the maturation of many European aged cheeses. The most common types of mold used in dairy production include *Penicillium*, *Geotrichum*, *Aspergillus*, *Mucor*, and *Fusarium*.^20,21^ Although many yeasts and molds are beneficial in dairy production, others are associated with milk spoilage.^20^

### 3.3 Interaction of LAB, yeast, and mold in fermented milk

In a well-balanced dairy system, the complex and diverse interactions of LAB, yeasts, and molds support the balanced growth and maintenance of beneficial microbial communities, while suppressing the growth of food spoilage microorganisms and potential pathogens, including *Clostridium botulinum*, *Staphylococcus aureus*, and *Listeria monocytogenes*.^20,57^ The growth of LAB produces fermentation end products that serve as a major energy source for yeasts and molds. For example, galactose is a byproduct of lactose metabolism by most lactobacilli, and it serves as a carbon source for the growth of non-lactose fermenting yeasts.^53^ At the same time, the release of free amino acids such as leucine, phenylalanine, lysine, arginine, glutamic acid, and valine during yeast metabolism promotes the growth of LAB within low proteolytic systems and provides substrates for the synthesis of secondary metabolites.^65^ Moreover, yeast synthesis of other compounds such as carbon dioxide, pyruvate, propionate, succinate, and vitamins can also be beneficial for LAB growth.^66^

In addition to supporting each other's growth, LAB and yeasts also contribute to mutual defense. Their combined fermentation byproducts, which include lactic acid, ethanol, and carbon dioxide, inhibit the growth of competing spoilage species, and some LAB and yeasts are further able to directly inhibit the growth of undesired microbes through the production of antimicrobial peptides.^67^*Lactococcus lactis*, for example, produces the bacteriocin nisin, a well-characterized antimicrobial peptide that is used as a natural food preservative due to its ability to inhibit the growth of many Gram-positive bacteria, as well as *Clostridium* and *Bacillus* spores.^68^ The cyclic lipopeptide iturin V from *Lactobacillus* sp. M31 likewise prevents food spoilage by inhibiting the growth of pathogenic bacteria, including *P. aeruginosa* and *Vibrio cholerae*.^69^ Some dairy yeasts are also known to produce antimicrobial proteins, such as mycocin-producing strains of *D. hansenii*.^66^

**Fig. 3:**
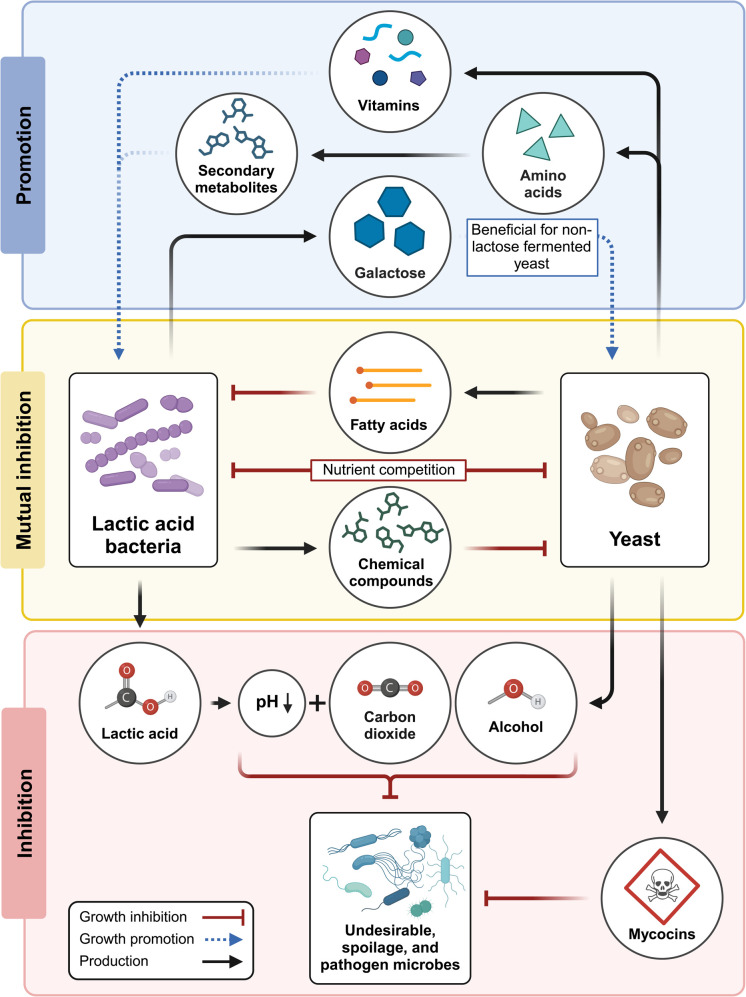
Complex interaction between lactic acid bacteria (LAB) and yeast in milk results in growth promotion and/or inhibition. Created with BioRender.

Within dairy systems, however, there may also be carbon source competition among beneficial microbes, as well as the production of compounds that inhibit LAB or yeast growth. For example, compounds produced by LAB such as phenyl-lactic acid, 4-hydroxy-phenyl-lactic acid, and cyclic peptides can inhibit the growth of dairy yeasts, and certain bacterial enzymes can lyse the yeast cell walls. Likewise the growth of LAB may be inhibited by fatty acids produced by lipolytic yeasts. The production of proteases and lipases by yeasts during the degradation of milk proteins and fats can also lead to spoilage of dairy products and cause excessive gas formation, off-flavors, coagulation, or discoloration.^28,66,70^ Consequently, the delicate balance between LAB and yeasts within dairy ferments is very important for maintaining dairy product fidelity and stability. Fig. 3 summarizes the complex interaction between LAB and yeast in milk.

Milk is a nutrient-rich fluid that provides optimal conditions not only for the growth of technologically relevant and health-promoting microorganisms, but also for spoilage microbes and pathogens.^20^ The consumption of raw milk products containing *Salmonella*, *Shigella*, shiga toxin-producing *Escherichia*, *Campylobacter*, *Brucella*, or *Listeria* species is hazardous to health and a major source of foodborne illness.^20,71^ Numerous epidemiological outbreaks of these pathogens have been documented as a result of the consumption of raw milk contaminated either from an infected dairy animal or improperly cleaned dairy processing equipment.^72,73^

After milk collection, the composition of dairy microbiota can change substantially depending on storage conditions. Cold storage and refrigeration prior to consumption or further processing is known to shift the microbial profile towards psychrotrophic bacteria that can grow at temperatures of 3–7 °C, while suppressing the growth of beneficial microbes, such as LAB. Raw milk microbiota naturally contains psychrotrophic taxa, such as *Bacillus*, *Acinetobacter*, and *Pseudomonas*, and it is the overgrowth of these taxa during cold storage that generally determines the shelf life of refrigerated dairy products.^74,75^ Among the psychrotrophic bacteria, *Pseudomonas* is most frequently isolated from raw milk, making up 65–70% of all isolated psychrotrophs.^28,76^ In addition to their natural occurrence, *Pseudomonas* can easily contaminate milk during collection, transport and processing. *Pseudomonas* spp. have also been detected in traditional fermented dairy products such as *airag* from Mongolia.^77,78^ The following paragraphs discuss the specific niche adaptation of *Pseudomonas* to milk.

## 4 *Pseudomonas* in dairy systems

The genus *Pseudomonas* was first described by Migula in 1894 and is a highly diverse genus belonging to the Pseudomonadaceae family within the Gammaproteobacteria class of bacteria. These bacteria are Gram-negative, aerobic, non-fermentative, catalase and oxidase-positive, and typically occur as non-spore-forming rods. Pseudomonads are mesophilic but also exhibit psychrotolerant characteristics, allowing them to grow across a temperature range of 4–42 °C.^76,79^ Pseudomonads have relatively simple nutritional requirements and are highly adaptable to various conditions, allowing them to inhabit a wide range of environments and ecological niches and modify their metabolism based on environmental conditions. They are commonly found in soil, sediments, water (both freshwater and marine environments), air, plants, fungi, and algae, as well as in association with humans and animals. Additionally, they have been isolated from diverse manufactured sources, including processed foods, clinical instruments, aseptic solutions, cosmetics, and medical products.^80,81^ The most common *Pseudomonas* species identified in milk and dairy products are *P. fluorescens*, *P. gessardii*, *P. fragi*, and *P. lundensis*.^82^*P. putida*, which is also found in milk, alters its metabolic pathways depending on temperature; 266 genes, representing about 5% of its genome, were found to be differentially expressed in response to temperature changes from 30 °C to 10 °C. These genes are involved in energy metabolism, transport and binding of substrates (particularly related to the uptake of amino acids), and other essential cellular functions. At 10 °C, there is an increased uptake and metabolism of amino acids such as proline, valine, leucine, isoleucine, phenylalanine, tyrosine, and glutamine, as well as increased glucose uptake. The metabolic changes appear to minimize problems that occur at low temperatures, such as protein misfolding or reduced membrane fluidity.^83^ The ability to thrive in a huge diversity of niches combined with efficient cold adaptation of *Pseudomonas* facilitates the colonization of this genus in milk.

### 4.1 Relevance of *Pseudomonas* for shelf life of milk

*Pseudomonas*, as a predominant genus in raw milk, has attracted attention in studies of the shelf life of both refrigerated and heat-treated dairy products due to its rapid proliferation at low temperatures and its production of heat-resistant spoilage enzymes. It is predominantly the action of its extracellular lipases and proteases that leads to the spoilage of milk and dairy products in cold storage.^28,84^*Pseudomonas* spoilage enzymes are difficult to eliminate from dairy systems, as many are heat-resistant and able to retain activity after pasteurization (72 °C for 15 s) or ultra-high temperature treatments (135–150 °C for 2–10 s)^56^ and they are the main factor limiting the shelf life and quality of ultra-high temperature (UHT) milk.^81^ The most important *Pseudomonas* peptidase is the heat-resistant alkaline metallopeptidase AprX (also known as AprA^85^) which has been identified in several strains of *Pseudomonas.*^82^ The gene *aprX* was found to be highly conserved among *Pseudomonas* species isolated from raw milk but showed heterogeneity in proteolytic activity. The caseinolytic potential of AprX from different *Pseudomonas* strains is variable and can hydrolyze all four milk caseins, but especially β-casein and κ-casein. This leads to an increase in viscosity and causes a bitter taste and gelation of milk, which further limits shelf life.^29^ The proteolytic activity of AprX is linked to the lipolytic activity of LipA, as the genes for the protease and the lipase are located on the same operon. This lipase is involved in producing rancidity and a soapy off-flavor due to the hydrolysis of milk fat.^29^

**Fig. 4:**
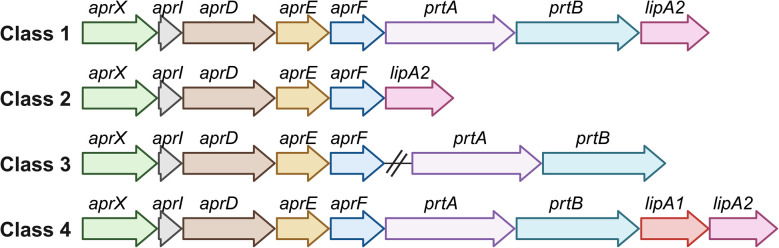
Genetic organization of the most common classes of the *aprX-lipA2* operon in *Pseudomonas*. Adapted from Maier *et al.*^85^ Created with BioRender.

The *aprX-lipA2* operon (Fig. 4) usually contains six to nine genes that code for different components, including a metallopeptidase (*aprX*), a peptidase inhibitor (*aprI*), a type I secretion system (*aprD*, *aprE* and *aprF*), two potential autotransporter homologs (*prtA* and *prtB*), and two lipases (*lipA1* and *lipA2*).^85^ However, there are strain-specific variations in the genetic organization of the *aprX-lipA2* operon. A total of 22 different types have been identified, with most of the variations affecting the last four genes; these genes vary both in their presence and in their arrangement within the operon.^85^

During cold storage, *Pseudomonas* shows reduced AprX activity and a slower growth rate, which is probably compensated by increased AprX production as part of its survival strategy. By synthesizing heat-resistant peptidases, *Pseudomonas* ensures the degradation of proteins even under difficult conditions, enabling efficient nutrient utilization and continued proliferation.^29^ The proteolytic potential of *Pseudomonas* species in milk is closely linked to the presence and arrangement of genes within the *aprX-lipA2* operon. Specifically, strains that possess the complete operon structure (Fig. 4 class 1) exhibit significantly higher proteolytic activity compared to those with variations or deletions in this gene cluster.^85^ Genetically modified *Pseudomonas* strains lacking the *aprX-lipA2* operon may provide potentially useful applications in industrial processes such as fermentation. To the best of our knowledge, this specific aspect has not been addressed by other researchers and could be further investigated. However, it has been shown that *Pseudomonas* strains isolated from milk can exhibit proteolytic activity even in the absence of the *aprX* gene.^86^

In addition to producing heat-resistant peptidases, *Pseudomonas* also has the ability to form biofilms under various environmental conditions. These biofilms can colonize surfaces of equipment, cow udders, or silage on the farm, making it easy for *Pseudomonas* to contaminate milk. Biofilms are a common problem in the dairy industry. Once they have formed, they are difficult to remove during the milk processing, which is a major problem for maintaining hygiene and preventing contamination.^87,88^ Besides the negative impact of *Pseudomonas* on the shelf life of milk and dairy products, the great potential of pseudomonads to produce antimicrobial natural products helps to eliminate other spoilage bacteria and prevent premature spoilage.

### 4.2 *Pseudomonas* and its natural product potential

The genus *Pseudomonas* currently consists of 330 named species (as of March 2024) divided into 13 phylogenetic groups,^89^ which likely represents a fraction of the true biological diversity of the genus.^90^ Species with validated names are provided in the List of Prokaryotic Names with Standing in Nomenclature (LPSN) available at http://www.bacterio.net.^91^ The genomic diversity of *Pseudomonas* is vast. So far, 1324 complete genomes and 12 906 draft genomes of *Pseudomonas* strains have been published in the *Pseudomonas* Genome Database (http://www.pseudomonas.com).^92^ The genomes of *Pseudomonas* species range between 3 and 7 Mbp in size, and have approximately 3000–7000 putative genes.^37^ These genomes contain a large number of biosynthetic gene clusters (BGCs), which are responsible for the production of a wide range of secondary metabolites, including natural products such as nonribosomal peptides (NRPs), polyketides (PKs), nonribosomal peptide/polyketide hybrids (NRP/PK hybrids), ribosomally synthesized and post-translationally modified peptides (RiPPs), alkaloids, and terpenoids.^93^

The genome of *P. batumici* UCM B-321^T^ contains the highest number of reported BGCs (23), whereas *P. caeni* DSM 24390^T^, which has the smallest reported genome, contains only one.^37^ In total, *Pseudomonas* species carry on average 6–16 BGCs per genome. However, many BGCs are not expressed under laboratory conditions and are known as silent gene clusters.^93^

**Fig. 5:**
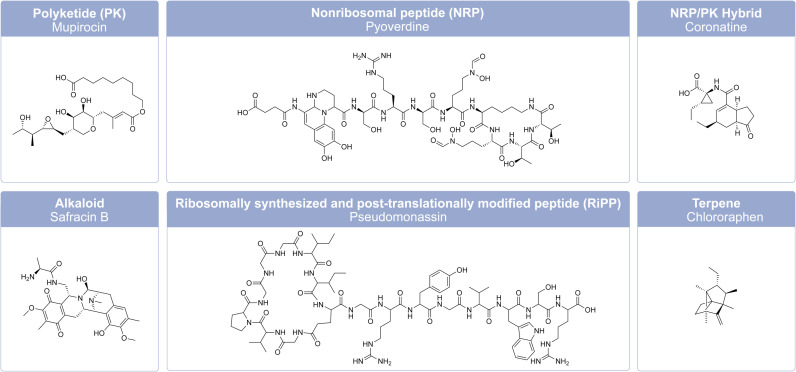
Example of chemical structures of six major classes of secondary metabolites from *Pseudomonas*. Polyketide (PK) mupirocin from *P. fluorescens* NCIMB 10586,^91^ nonribosomal peptide (NRP) pyoverdine from *P. aeruginosa* PAO,^34^ nonribosomal peptide/polyketide hybrid (NRP/PK hybrid) coronatine from *P. syringae* pv. glycinea PG4180,^34^ alkaloid safracin B from *P. fluorescens* A2-2,^34^ ribosomally synthesized and post-translationally modified peptide (RiPP) pseudomonassin from *Pseudomonas* sp. SST3,^92^ and terpene chlororaphen from *P. chlororaphis* O6.^93^

Fig. 5 presents examples of representative *Pseudomonas*-derived natural products from each of the six classes. The majority of BGCs in *Pseudomonas* code for nonribosomal peptide synthetases (NRPSs)^37^ that catalyze the synthesis of a variety of secondary metabolites, which include enzymes, siderophores, antibiotics, toxins or biosurfactants. Siderophores such as pyoverdines are known to be responsible for the anomalous discoloration of dairy products.^94^ In contrast, terpene and terpenoids are generally underrepresented in *Pseudomonas*. For example, in a recent genome mining study from Alam *et al.*^93^ it was found that terpene BGCs are only present in 3 of 37 *Pseudomonas* genomes. Many of the NRPS-derived compounds from *Pseudomonas* are cyclic lipopeptides (CLPs), which display potent antimicrobial, antitumor, or biosurfactant activities. They can also act as virulence factors or chemical signal molecules involved in quorum sensing.^35^

### 4.3 Nonribosomal lipopeptides from *Pseudomonas*

Nonribosomal lipopeptides (LPs) belong to the class of NRPs. They have a characteristic fatty acid chain connected to the N-terminal end of the peptide.^41^ NRPSs catalyze the production of both cyclic or linear LPs (CLPs or LLPs).^95^ The incorporation of the lipid moiety into the peptide backbone is catalyzed by a starter condensation (C_starter^−^_) domain, which is located within the initial module of the NRPS and initiates the *N*-acylation of the first amino acid, a process known as lipoinitiation.^41,96^ LPs are mainly produced by the genera *Bacillus*, *Pseudomonas*, and *Streptomyces*.^36^ However, this section only focuses on LPs from *Pseudomonas*.

**Fig. 6:**
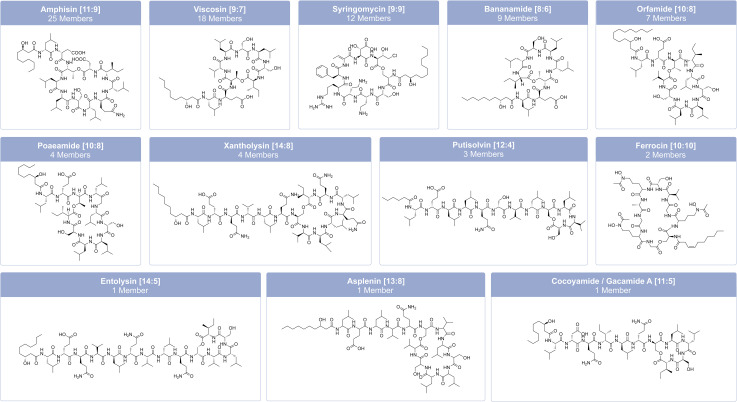
Family classification and chemical structures of short cyclic lipopeptides (CLPs) from pathogenic and non-pathogenic *Pseudomonas* strains. The characteristic combination of peptide length (*L*) and macrolactone ring size (*S*) is given in brackets after the family name as [*L*:*S*]. The individual member names from left to right: amphisin, viscosin, syringomycin E, bananamide A, orfamide A, poaeamide A, MA026, putisolvin I, ferrocin A, entolysin A, asplenin, and cocoyamide or gacamide A.

**Fig. 7:**
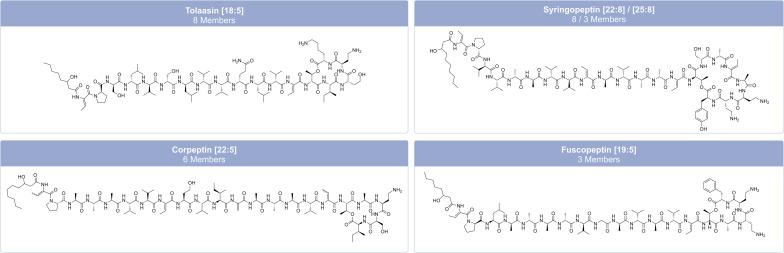
Family classification and chemical structures of long cyclic lipopeptides (CLPs) from pathogenic and non-pathogenic *Pseudomonas* strains. The characteristic combination of peptide length (*L*) and macrolactone ring size (*S*) is given in brackets after the family name as [*L*:*S*]. The individual member names from left to right: tolaasin I, SP22-A, corpeptin A and fuscopeptin A.

The structural diversity of LPs includes variations in oligopeptide length from 8–25 amino acids, amino acid-configurations, fatty acyl residue length from C_5_ to C_16_, and cyclic LPs with macrocycles of 4–9 amino acids.^34,43^ Most LPs from *Pseudomonas* consist of a limited number of proteinogenic amino acid substrates, including hydrophobic amino acids (Leu, Val, and Ile), polar amino acids (Ser, Thr and Gln), acidic amino acids (Glu and Asp), and a basic amino acid (Lys).^95^ Nevertheless, they can also contain modified and non-proteinogenic amino acids, such as 2,4-diaminobutyric acid (Dab) and 4-chloro-threonine, as in syringomycin (Fig. 6), or 2,3-dehydroaminobutyric acid (Dhb) and homoserine (Hse), as in tolaasin (Fig. 7).^41,95^

**Fig. 8:**
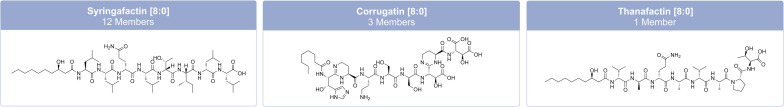
Family classification and chemical structures of linear lipopeptides (LLPs) from pathogenic and non-pathogenic *Pseudomonas* strains. The characteristic combination of peptide length (*L*) and macrolactone ring size (*S*) is given in brackets after the family name as [*L*:*S*]. The individual member names from left to right: syringafactin A, corrugatin, and thanafactin A.

*Pseudomonas*-derived LPs are classified into distinct families based on their amino acid sequence, length, and the number of amino acids within the macrolactone ring.^95^ Focusing only on non-pathogenic *Pseudomonas* strains, Cesa-Luna *et al.*^95^ have identified at least 13 LP-families, which include both CLPs (viscosin, bananamide, orfamide, poaeamide, amphisin, cocoyamide or gacamide,^97^ putisolvin, asplenin, xantholysin, entolysin, and tolaasin) and LLPs (syringafactin and thanafactin).^95^ We divide lipopeptides into 18 groups, distinguishing between short CLPs (Fig. 6), long CLPs (Fig. 7), and LLPs (Fig. 8), and identify additional families, such as syringomycin, syringopeptin (SP22 and SP25), ferrocin, fuscopeptin, corrugatin, and corpeptin, when including pathogenic *Pseudomonas* strains.^35^ In total, this results in 20 characterized LP families within pathogenic and non-pathogenic *Pseudomonas* strains. Some LPs remain unclassified^35^ and therefore belong to an additional provisional group.

Within these families, there is limited variation in both the fatty acid composition and the amino acid sequence and/or configuration of the individual amino acids within the peptide.^95^ Usually, the lipid moiety is a β-hydroxy acid.^35^ Viscosin and amphisin are the most abundant families with 25 and 18 members, respectively (Fig. 6).^95^ LPs of the viscosin group typically consist of 9 amino acids, while LPs of the amphisin group contain 11 amino acids. Known members of the amphisin group differ in three of their amino acids at positions 8, 9, and 11. Both groups commonly contain a 3-hydroxydecanoic acid (HDA) tail.^40^ Milkisin is a member of the amphisin family and was isolated from the milk-associated *Pseudomonas* sp. UCMA 17988, and differs from amphisin by having an Asp as the last amino acid (Fig. 6), whereas milkisin contains Glu (HDA-Leu_1_-Asp_2_-Thr_3_-Leu_4_-Leu_5_-Ser_6_-Leu_7_-Gln_8_-Leu_9_-Ile_10_-Glu_11_). The three congeners of milkisin differ only in the length of their fatty acid moieties.^98^

Structurally similar CLPs can also be found in other environmental *Pseudomonas* species, *e.g.*, stechlisin from *Pseudomonas* sp. FhG100052 ^99^ isolated from freshwater and tensin from *P. fluorescens* 96.578 isolated from sugar beet rhizosphere soil.^100^ The two *Pseudomonas* strains P866 and P867 isolated from raw milk secreted four lipodepsipeptides that clearly fall within the viscosin group. The two major compounds have molecular weights of 1168.7 and 1140.7 Da, respectively. Their amino acid sequence is Leu_1_-Glu_2_-Thr_3_-Ile_4_-Leu_5_-Ser_6_-Leu_7_-Ser_8_-Ile_9_, differing only in their fatty acid moiety with 3-hydroxydodecanoic acid (HDDA) and 3-hydroxydecanoic acid (HDA).^101^ To the best of our knowledge, *Pseudomonas* sp. UCMA 17988 and the strains P866 and P867 are the only strains isolated from milk reported to produce CLPs. LPs of the tolaasin group exhibit a greater diversity, characterized by peptide chains with 19–25 amino acids and variations of the lipid tails.^40^

Often, a single biosynthetic NRPS cluster produces more than one CLP due to an assumed flexibility of some adenylation domains that are responsible for amino acid recognition.^36^ While mono-producers secrete LPs (one or more congeners) belonging to a specific chemical family (*e.g.*, *P. fluorescens* strain SS101 produces at least eight structural analogs of massetolide A^41^), dual producers can synthesize CLPs from two distinct families, such as tolaasin and pseudodesmin in *P. tolaasii*.^102^

### 4.4 Biological properties of nonribosomal lipopeptides from *Pseudomonas*

**Fig. 9:**
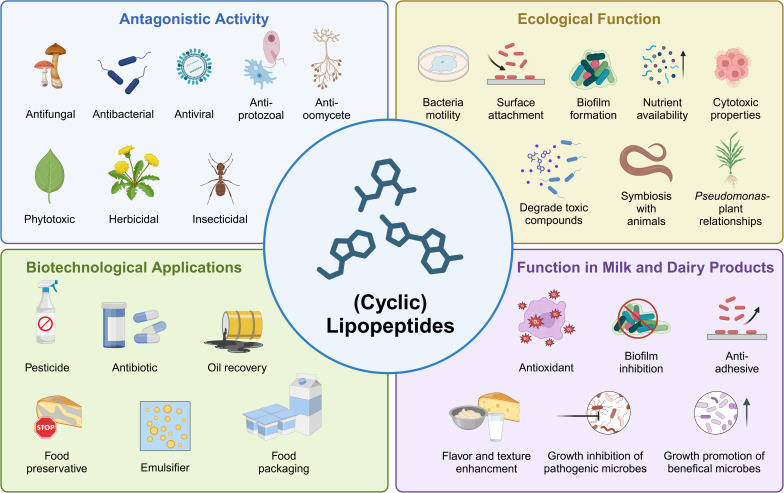
Overview of antagonistic activities, different functions, and applications of LPs. They exhibit a broad spectrum of antimicrobial activities including antibacterial, antifungal, anti-oomycete, antiprotozoal, antiviral, phytotoxic, herbicidal, and insecticidal activities. They promote bacterial mobility and are involved in surface attachment and biofilm formation. LPs play an important role in ecological interactions with plants or other organisms. Some LPs enhance the nutrient availability and degradation of toxic compounds and possess cytotoxic properties. It is beneficial in milk and dairy products that LPs have antioxidative and antiadhesive properties and can prevent biofilms. LPs inhibit the growth of pathogens in milk and favor the growth of beneficial microbes, while also enhancing the flavor and texture of dairy products. Due to their wide spectrum of activities and functions, LPs have many applications in the pharmaceutical and food industries. Created with BioRender.

Due to their great structural diversity, LPs exhibit a wide range of biological functions and properties that are crucial for microbial survival and ecological interactions (Fig. 9).^43^ LPs contribute to the ecological success of *Pseudomonas* by enhancing survival under challenging conditions and facilitating interactions with other microorganisms and plants, as they enable *Pseudomonas* to defend itself against competitors and predators.^35,36^ This is mainly attributed to the antimicrobial activity of LPs (described in more detail below). LPs can enable *Pseudomonas* to thrive in dairy environments by inhibiting the growth of milk-associated microorganisms. In *Pseudomonas*-plant interactions, LPs can either promote plant health by acting as biocontrol agents that protect against disease (*e.g.*, keanumycin from *Pseudomonas* sp. QS1027 controls *Botrytis* blight caused by the phytopathogen *Botrytis cinerea*^103^) or cause plant disease by acting as phytotoxins and virulence factors (*e.g.*, cichopeptins from the lettuce midrib rot pathogen *Pseudomonas cichorii* SF1-54 cause leaf necrosis^104^). Plant pathogenic pseudomonads usually colonize the leaf surface, whereas biocontrol pseudomonads inhabit the rhizosphere.^105^

LPs are amphiphilic due to the presence of a hydrophilic peptide-based head and lipophilic fatty acid tail.^34,35^ As a consequence, LPs can act as biological surfactants lowering surface tension and hence promoting the active movement of bacteria. Bacterial mobility can occur through a range of mechanisms, including swarming, swimming, twitching, gliding, and sliding.^36^ Cellular swarming promotes the distribution of bacteria in plants and facilitates attachment and colonization of surfaces.^43^ LPs not only play an important role in surface attachment but also in the formation and development of biofilms.^41^ While some LPs, like xantholysin,^106^ sessilin,^105^ and viscosin^107^ contribute to the formation of biofilms, others like arthrofactin,^108^ orfamide,^105^ and putisolvin^109^ prevent biofilm formation. Rossi *et al.*^110^ demonstrated that not all milk-associated *Pseudomonas* strains were able to produce biofilms and that production is temperature dependent. Low temperatures (<10 °C) promote biofilm formation. They have also shown that biofilm formation is correlated with the production of blue pigment by *P. fluorescens*.^110^

The physicochemical properties of LP enable pseudomonads to acquire nutrients due to an increase in the bioavailability of water-insoluble substrates^34^ while also degrading toxic compounds.^35,41^

In addition to their physicochemical characteristics, LPs also have notable biological properties. Most LPs, particularly the cyclic LPs, display potent antagonistic activities against a broad range of organisms including bacteria, fungi, oomycetes, protozoa, viruses, insects, nematodes, and plants.^34–36,41,42^ The antagonistic activities of LPs can be used in the food and dairy industry to delay food spoilage. Examples of potential food preservatives include the non-*Pseudomonas*-derived linear lipotridecapeptides brevibacillin V (FA-Dhb_1_-Leu_2_-Orn_3_-Ile_4_-Val_5_-Val_6_-Lys_7_-Val_8_-Val_9_-Lys_10_-Tyr_11_-Leu_12_-Valinol_13_) and brevibacillin (FA-Dhb_1_-Leu_2_-Orn_3_-Ile_4_-Ile_5_-Val_6_-Lys_7_-Val_8_-Val_9_-Lys_10_-Tyr_11_-Leu_12_-Valinol_13_) that effectively kill the pathogens *Staphylococcus aureus* and *Listeria monocytogenes* in skim milk.^111^ Similarly, the *Pseudomonas*-derived CLP milkisin prevents spoilage of milk by inhibiting the growth of *S. aureus* CIP 53.154 and *Salmonella enterica* Newport CIP 105629 in raw milk.^98^

The CLPs from *Pseudomonas* strains P866 and P867 showed antimicrobial activity against milk contaminants and spoilage organisms *S. aureus*, *Bacillus cereus*, and *Bacillus subtilis* and inhibitory activity against the most commonly used test organism in microbiological inhibitor assays (MIAs) *Geobacillus stearothermophilus* var. calidolactis.^101^ The antimicrobial activity of the CLPs can prevent milk spoilage, but can also interfere with MIAs and lead to false positive results, making it more challenging to detect actual antibiotic residues in raw milk.

LPs primarily exert their antimicrobial effects by interacting with cell membranes, disrupting their integrity and causing pore formation. This disruption results in an influx of H^+^ and Ca^2+^ ions and an efflux of K^+^ ions, which breaks the pH gradient across the membrane and ultimately leads to cell death.^42^ LPs additionally possess cytotoxic, antitumor, antiproliferative, and immunosuppressive properties.^36,40^ Such biological activities depend on the LP family, and even on the specific LP itself. For example, LPs of the mycin family show greater hemolytic activity, while LPs of the peptin family display stronger phytotoxicity.^43^

Beyond their antimicrobial effects, LPs can also promote microbial growth in some cases. For example, *B. licheniformis* MS48 from marine sponges produces a lipopeptide that enhances the growth of the probiotic LAB *L. delbrueckii* subsp. *bulgaricus* and *S. thermophilus*, which improves the flavor profile and shelf life of yogurts. This effect may be due to the release of free peptides and amino acids during the LP degradation of milk proteins, which the LAB can then utilize for growth. This LP has also been found to improve the survivability, tolerance, and growth of probiotic bacteria in harsh environments resembling the gastric environment (*e.g.*, in acid and bile), and cooking (high heat), making it suitable for food applications.^112^ Another example is a biosurfactant with an unknown structure but structural similarities to CLPs isolated from corn sour liquor (CSL), which was found to positively affect the growth of *L. casei* in drinking yogurt. This biosurfactant improves the functional properties of probiotic foods by promoting the growth of probiotic bacteria.^113^

## 5 Conclusion

Dairy products are a highly nutrient-rich environment that supports the development of a niche-specific microbiome. Its members play a diversity of roles that range from facilitating fermentation, modulating probiotic activity, and preventing and causing spoilage. The understanding of the dynamic and complex interaction within the dairy microbiome is important to control the quality and safety of dairy products for human consumption but also to use it for applications in pharmaceutical and food industries. Beyond typical milk-associated LAB, yeasts, and molds, many dairy products also support the growth of prolific secondary metabolite producers, such as psychrotrophic members of the genus *Pseudomonas*. Today, *Pseudomonas* is most associated with refrigerated dairy products, but it has likely also played a long-term role in the production and storage of traditional dairy products in cold climate regions, such as Mongolia. We provide a historical perspective on the evolution and use of dairy products, with a special emphasis on Mongolia, and give insights into how microorganisms have shaped traditional and modern dairy practices. Special attention is given to the impact of microbially produced natural compounds in dairy products, with a focus on *Pseudomonas*-derived natural products such as lipopeptides. Overall, the study of natural products from dairy-associated bacteria holds great promise to find novel natural products that could improve health or enhance food quality.

## 6 Data availability

No primary research data or results have been generated or analysed as part of this review.

## 7 Conflicts of interest

There are no conflicts to declare.
